# Effect of Laminin Derived Peptides IKVAV and LRE Tethered to Hyaluronic Acid on hiPSC Derived Neural Stem Cell Morphology, Attachment and Neurite Extension

**DOI:** 10.3390/jfb11010015

**Published:** 2020-03-06

**Authors:** T. Hiran Perera, Xi Lu, Laura A Smith Callahan

**Affiliations:** 1 Vivian L. Smith Department of Neurosurgery, McGovern Medical School at the University of Texas Health Science Center at Houston McGovern Medical School, Houston, TX 77030, USA; Thuduwage.H.Perera@uth.tmc.edu (T.H.P.); xi.lu@uth.tmc.edu (X.L.); 2 Center for Stem Cell and Regenerative Medicine, Brown Foundation Institute of Molecular Medicine, McGovern Medical School at the University of Texas Health Science Center at Houston, Houston, TX 77030, USA; 3 Graduate School of Biomedical Sciences, MD Anderson Cancer Center UTHealth, Houston, TX 77030, USA

**Keywords:** neural tissue engineering, human pluripotent stem cells, laminin, fibronectin

## Abstract

Low neural tissue extracellular matrix (ECM) content has led to the understudy of its effects on neural cells and tissue. Hyaluronic acid (HA) and laminin are major neural ECM components, but direct comparisons of their cellular effects could not be located in the literature. The current study uses human-induced pluripotent stem-cell-derived neural stem cells to assess the effects of HA, laminin, and HA with laminin-derived peptides IKVAV and LRE on cellular morphology, attachment, neurite extension and ECM remodeling. Increased attachment was observed on HA with and without IKVAV and LRE compared to laminin. Cellular morphology and neurite extension were similar on all surfaces. Using a direct binding inhibitor of Cav2.2 voltage gated calcium channel activity, a known binding partner of LRE, reduced attachment on HA with and without IKVAV and LRE and altered cellular morphology on surfaces with laminin or IKVAV and LRE. HA with IKVAV and LRE reduced the fluorescent intensity of fibronectin staining, but did not alter the localization of ECM remodeling enzymes matrix metalloprotease 2 and 9 staining compared to HA. Overall, the data indicate HA, IKVAV and LRE have complementary effects on human-induced pluripotent stem-cell-derived neural stem cell behavior.

## 1. Introduction

Due to its relatively low content in neural tissue [1], the effects of the extracellular matrix (ECM) on neural cell and tissue function have been understudied. However, recent evidence indicates that a number of ECM properties, including biochemical composition, have significant effects on the behavior and function of neural cell types and tissue [2,3,4]. This has significant ramifications for the development of biomaterials to support therapeutic and in vitro models of the central nervous system (CNS). Understanding how these changes in the ECM affect cellular response to the materials is necessary to effectively develop biomaterial formulations for the CNS.

Hyaluronic acid (HA) is a major component of the CNS ECM [5] that has been used as a backbone polymer for the development of matrices for neural stem cell growth and differentiation [6,7]. HA also stimulates matrix metalloprotease (MMP) 2 expression [8,9]. MMP 2 is associated with axonal regeneration after CNS injury [10,11,12] and ECM remodeling, which is emerging as an important regulator of neural cell behavior and tissue formation [4,13]. The addition of laminin to HA further enhances axon extension [14]. Laminin is a major constituent of the basement membrane that is often used in two dimensional (2D) neural cell culture. Direct comparisons of cellular response to HA and laminin surfaces were not found in the literature, and mixed results have been reported about neural cell response to both HA and laminin when compared with other molecules in 2D culture [15,16,17,18]. Understanding how each molecule contributes to cellular response would be beneficial in the rational development of biomaterial supports for neural cultures.

MMP 2 and 9, which HA-stimulates expression of [8,9], degrades laminin [19,20,21]. The degraded laminin fragments have a number of biological effects that intact laminin molecules do not possess [22]. Therefore, the utilization of bioactive peptides isolated from laminin will provide greater control of bioactive signal presentation than using the whole laminin molecule [22]. A number of laminin-derived peptides are known to have biological effects, and different peptides promote different cellular behaviors [22,23]. Laminin α1 chain derived Ile-Lys-Val-Ala-Val (IKVAV) promotes neural differentiation and axon extension [24,25] in addition to altering MMP 2 and 9 expression through calcium (Ca^2+^)-dependent integrin signaling mechanisms [26]. However, IKVAV does not always support cellular attachment [27,28]. Due to this and other shortcomings, additional peptides are often used to supplement IKVAV’s biological effects to achieve the desired biological response [29,30,31]. Leu-Arg-Glu (LRE), a peptide present in the laminin α2, β2 and γ1 chains [32], modulates Ca^2+^ flow through Cav2.2 voltage-gated Ca^2+^ channels, altering the expression of MMP 2 and 9 and inhibitors of MMP activity, called tissue inhibitors of metalloproteinases (TIMP) [33]. LRE further supports adhesion, axon guidance, and other cell behaviors not stimulated by IKVAV [32,34,35]. Our previous study of mouse embryonic stem cells encapsulated in HA matrices with IKVAV and LRE peptide signaling found increased axon extension due to altered MMP expression [36]. However, the cell response to peptide signaling can vary between species [37,38]. The present study probes the effects of IKVAV and LRE on human-induced pluripotent stem-cell-derived neural stem cells (hNSC), a potential cell source for CNS cell therapy treatments, in two-dimensional culture. IKVAV and LRE tethered to HA were found to support adhesion, matrix remodeling and neurite extension by hNSC 2D culture at a concentrations similar to those used with mouse embryonic stem cells.

## 2. Materials and Methods

**Materials and analytical instrumentation:** Supplies and chemicals were purchased from Thermo Fisher Scientific (Waltham, MA, USA) or VWR International (Radnor, PA, USA) unless specified otherwise. ^1^H spectra (Bruker, Billerica, MA, USA) were recorded at 600 MHz in D_2_O and were referenced to the residual proton. The degree of functionalization was probed by NMR analysis and normalized to the integral of acetyl–methyl signal at 2.041 ppm on the HA backbone.

**Peptide Synthesis:** Leu-Arg-Glu-Gly-Gly-Gly-Cys (LREGGGC, MW = 691.2 g mol^−1^), Acryl-Gly-Ill-Lys-Val-Ala-Val (IKVAV, MW = 640.3 g mol^−1^) and KCGPQGIWGQCK (GPQGIWGQ, MW = 1340.53 g mol^−1^) peptides were synthesized with a Biotage Initiator+Alstra automated microwave peptide synthesizer (Charlottesville, VA, USA) and purified with Biotage Isolera/Dalton2000 flash purification system as previously described [36,39]. Ultraviolet (254 nm) tracing from the Biotage Isolera/Dalton2000 flash purification system after mass-based sorting and mass spectra from an Advion Mass express ESI-MS spectrometer (Ithaca, NY, USA) were used to determine peptide purity. IKVAV and GPQGIWGQ were used without further processing, while dibenzocyclooctyne (DBCO)-maleimide was bound to LREGGGC in phosphate-buffered saline (PBS) as previously described [36].

**Dual functionalization of HA with thiol and azide (DIFF-HA):** HA (average Mw = 75 kDa, Lifecore, Chaska, MN, USA) was functionalized in a manner similar to that previously described [7,36]. Then dialyzed (MWCO = 12–14 kDa) against sodium chloride (1 M, 1 L) for 1 day, followed by dialysis against DDW (3 L) for the next 5 days with the DDW being changed daily. The product was frozen at −80 °C and lyophilized to obtain a white powder. Based on the ^1^H-NMR spectra, ~14% of the HA backbone was functionalized with thiols. ^1^H-NMR spectra cannot be used to determine azide functionalization due to proton overlap with the HA backbone. Azide content was measured indirectly by quantification of DBCO binding and was determined to be ~4% of the HA backbone.

**Functionalization of DIFF-HA with IKVAV and LRE peptides (PEP-HA):** DIFF-HA was functionalized with IKVAV and LRE as previously described [36]. Briefly, IKVAV (1.024 mg) was added to 100 mg of DIFF-HA in PBS overnight then dialyzed (MWCO = 10 kDa) against DDW for 3 days. DBCO-Maleimide-LREGGGC (3 mg) was added to the solution and stirred overnight. The solution was dialyzed (MWCO = 10 kDa) in DDW for 3 days, then lyophilized to obtain a white powder. Based on the ^1^H-NMR spectra of the product, ~5% of the backbone remained thiol-functionalized, ~9% of the backbone was IKVAV-functionalized and ~4% of the backbone was LRE-functionalized.

**Surface coating fabrication and characterization:** Tissue culture plates were coated with poly-L-ornithine (20 µg/mL) at 4 °C overnight then washed with PBS. A 1% solution of DIFF-HA or PEP-HA or mouse laminin (20 µg/mL, Corning 354232) control was then allowed to absorb onto plate for 1 h, followed by three washes with PBS. The amount of DIFF-HA or PEP-HA deposited on the surface was quantified using Alcian blue quantification of glycosaminoglycan as previously described [40,41]. Briefly, culture surface was stained with 0.5% Alcian blue (Sigma) for 1 h and then washed with PBS and water. Samples were then destained twice in 3% acetic acid, washed in PBS then dye was extracted with 8 M guanidine HCl overnight. The supernatant was centrifuged and the absorbance read at 600 nm on a microplate reader (Tecan Infinite M1000, Maennedorf, Switzerland). A standard curve of HA, stained according to Alcian blue protocol above and pelleted by centrifuge for 10 min at 16,000× *g* at 4 °C, was used to determine HA amount. The DIFF-HA and PEP-HA surface coatings were found to contain 2.14 ± 0.19 µg/mL and 2.29 ± 0.36 µg/mL, respectively (n = 5).

**Human-Induced Pluripotent Stem Cell Derived Neural Stem Cell (hNSC) Culture:** hNSC derived from the ND2.0 human induced pluripotent stem cell line were isolated from neural rosettes after 10 days of neural differentiation from the pluripotent state according to a previously published protocol [42,43]. Similar to previous studies [37,39,44,45], hNSC were then expanded on Matrigel coated flasks in N2B27 maintenance media (50% F12/DMEM, 50% Neurobasal medium, 1% Glutamax, 1% non-essential amino acids, 0.5% N2 supplement, 1% B27 supplement, 1% penicillin/streptomycin and 20 ng/mL FGF-2). For 2D studies, 25,000 hNSC (passage 12) were plated in each well of a 24-well plate unless otherwise noted. Neural differentiation media (1:1 mixing ratio of neurobasal media: F12/DMEM media, 1X Glutamax, 1X N2, 1X B27, 1X non-essential amino acid, 1% pen/strep, 20 ng/mL brain-derived neurotrophic factor, 20 ng/mL Glial cell-line-derived neurotrophic factor, 200 ng/mL ascorbic acid, 500 ng/mL cyclic adenosine monophosphate) was used for differentiation studies. Culture media was completely changed every other day.

**Immunofluorescence:** At designated timepoints, samples were fixed with 4% paraformaldehyde for 20 min. A solution of 0.1% Tween X in PBS was then added for 30 min, followed by blocking with 5% donkey serum in PBS for 1 h to minimize nonspecific antibody binding. Samples were incubated with purified anti-neuron-specific class III β-tubulin (TUJ1, BioLegend san Diego, CA, USA, catalog #: 801201, 1:500), MMP 2 (catalog #: PA1-1667, 1:1000), MMP9 (Millipore Sigma, St. Louis, MO, USA, catalog #: AV33090, 1:1000), vinculin (Millipore Sigma, catalog #: V4505, 1:1000), phalloidin (catalog #: U0292, 1:2500), fibronectin (Millipore Sigma, catalog #: F3648, 1:5000), or collagen IV (Millipore Sigma, catalog #: AB769, 1:500) antibodies at 4 °C overnight and treated with appropriate fluorescently conjugated donkey anti-rabbit or anti-mouse IgG (1:1000) antibodies 4 °C overnight. Cell nuclei were stained with DAPI (1:1000). All the steps were followed by several washes of PBS. All the images were taken using an inverted fluorescence microscope (Nikon TE2000-E, Tokyo, Japan). The tracing of cellular-perimeters-based cytoskeletal staining (phalloidin or TUJ1) in ImageJ was used to calculate cellular area, aspect ratio (ratio of the cellular length to width) and circularity (4 × π × area/perimeter^2^) (n = 3 with greater than 80 cells analyzed per condition). The length of projection extension was defined as the distance from the termination of phalloidin-staining in projections of a cell body to the closest edge of the nucleus (n = 3 with over 400 projections analyzed per condition). Fluorescent intensity was measured using ImageJ by thresholding an image to create a region of interest and then applying that region of interest towards the original image. Then, the average pixel intensity per unit area was determined and compared between the different groups (n = 3 with at least seven images analyzed per sample). The percentage of cells stained positive for TUJ1 and polarization (organization of the more cytoskeleton on one side of the nucleus begin neurite formation) (n = 3 with over 400 cells analyzed) and the length of neurite extension defined as the distance from the termination of TUJ1 staining in projections of a cell body to the closest edge of the nucleus were measured (n = 3 samples with over 100 axons measured per formulation).

**CaV2.2-blocking studies of attachment and axon extension:** To assess the contribution of CaV2.2 to cellular attachment, hNSC in suspension were exposed to 0.4 ng/mL of ω-conotoxin GVIA or vehicle (ultrapure sterile water) in human N2B27 maintenance media for 20 min. hNSC (passage12) were then plated at 10^5^ cells per cell in 48-well plates coated with DIFF-HA, PEP-HA or control (poly-L-ornithine and mouse laminin (20 µg/mL, Corning 354232)-coated surface) and allowed to adhere for 48 h. The culture surface was washed with PBS to remove non-adherent cells and then adherent cells were removed with accutase. Harvested cells were treated with 0.1% tween X in PBS for 15 min and pelleted. The supernatant was removed and the pellet resuspended in PBS. A Quant-iT Pico Green dsDNA Fluorescence Kit was used to quantify the DNA content according to manufacturing protocols. To determine the contribution of CaV2.2 to axon extension in 2D culture, 25,000 hNSC were plated on the surface of a 48-well plate and allowed to attach for 48 h. ω-Conotoxin GVIA (0.4 ng/mL) or vehicle was then added to neural differentiation media. Media was changed every other day. After 7 days, cells were fixed and stained for TUJ1 expression as described above.

**Statistics**: All quantitative data are presented as mean ± standard deviation of the mean. Two-way ANOVA followed by Bonferroni’s multiple comparison post hoc tests were conducted where appropriate using GraphPad Prism version 5.01 (GraphPad Software, La Jolla, CA, USA). Comparisons between two groups were evaluated using an unpaired two-tailed student *T*-test to determine significance. A *p*-value of less than 0.05 in all analysis determined significance. All experiments had at least two independent replicates. The number of independent replicates with a technical replicate number for each experiment is as follows: phalloidin and vinculin staining had three independent replicates with one technical replicate per experiment; adhesion staining had two independent replicates with three technical replicates per experiment; TUJ1 staining had two independent replicates with two or one technical replicates per experiment, MMP staining had three independent replicates with one technical replicate per experiment and fibronectin staining had two independent replicates with three technical replicates per experiment. A final n ≥ 3 was given for each timepoint. The minimum N for each experimental is contained in the description of the methods for the experiment.

## 3. Results

**Figure 1:**
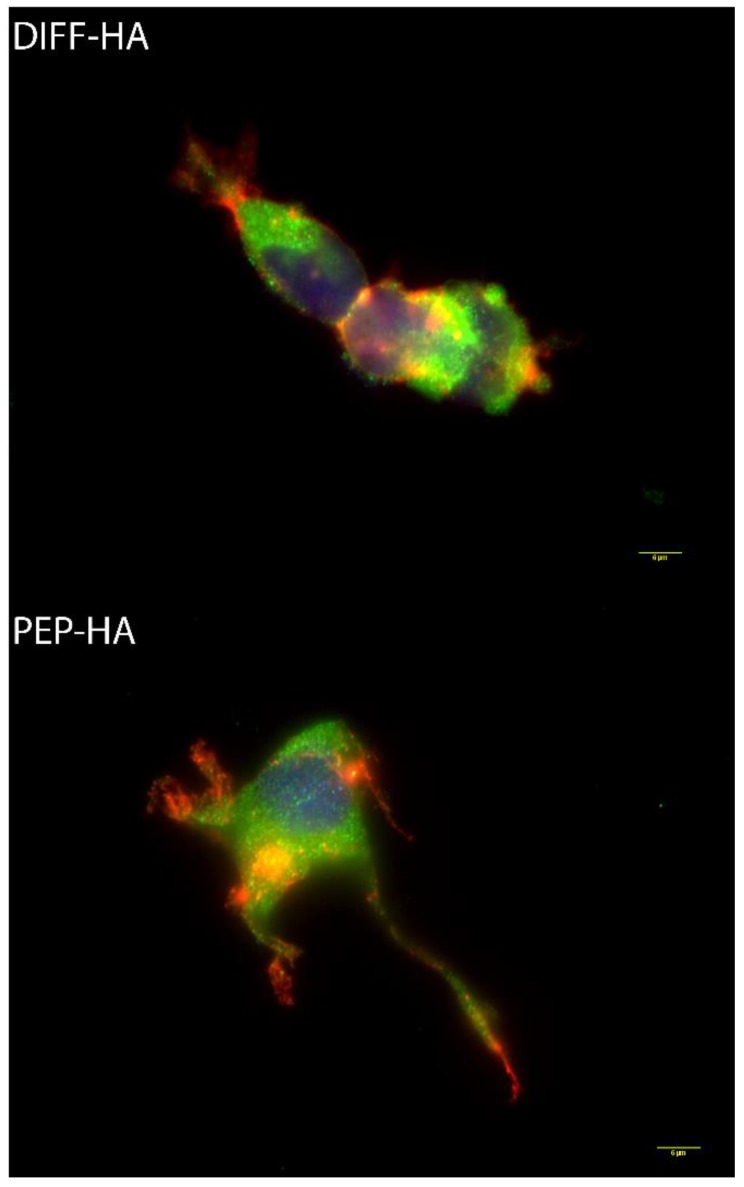
Phalloidin cytoskeletal staining (red) and vinculin staining (green) with nuclear staining (blue) of hNSC after 48 h of culture in N2B27 maintenance media on hyaluronic-acid-coated tissue culture plastic with (PEP-HA) and without (DIFF-HA) IKVAV and LRE peptide signaling. Scale bar = 6 µm.

Cytoskeletal staining 48 h after hNSC plating on thiol and azide di-functionalized HA (DIFF-HA) and IKVAV and LRE peptides tethered to HA (PEP-HA)-coated surfaces do not indicate significant morphological differences (Figure 1, cellular area: DIFF-HA = 1101.4 ± 371.4 µm^2^ and PEP-HA = 1024.6 ± 470.5 µm^2^, circularity: DIFF-HA = 0.39 ± 0.06 and PEP-HA = 0.34 ± 0.08, aspect ratio: DIFF-HA = 2.56 ± 0.52 and PEP-HA = 2.86 ± 0.18, projection number DIFF-HA = 1.95 ± 0.33 and PEP-HA = 2.43 ± 0.41 and projection length: 19.8 ± 7.2 µm and PEP-HA = 23.9 ± 6.7 µm, N ≥ 80 cells and 400 projections from three independent samples). hNSC culture on a laminin control surface yielded a similar cytoskeletal morphology to that of DIFF-HA and PEP-HA (Supplemental Figure S1).

**Figure 2:**
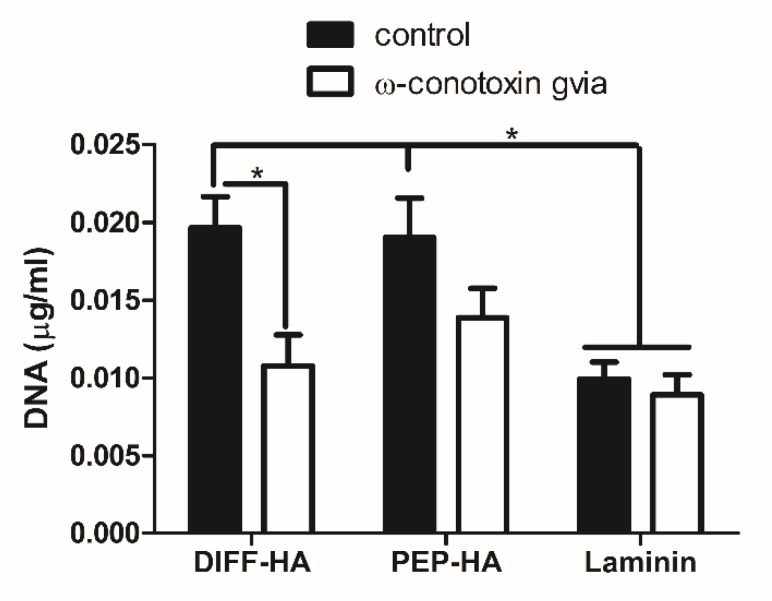
Attachment of hNSC with hyaluronic acid coated tissue culture plastic with (PEP-HA) and without (DIFF-HA) IKVAV and LRE peptide signaling or a laminin-coated control surface 48 h after plating. Small molecule ω-conotoxin GVIA was used to block calcium signaling through the CaV2.2 voltage gated Ca^2+^ channel, a known binding target of LRE. * indicates *p*-value < 0.05.

Cellular attachment was similar between DIFF-HA and PEP-HA at the 48 h timepoint (Figure 2). The use of small molecule ω-conotoxin GVIA to block Ca^2+^ flow through Cav2.2 voltage-gated Ca^2+^ channels, a known binding partner for the LRE peptide, significantly reduced adhesion to the DIFF-HA surface, but not the PEP-HA or the laminin control surfaces (Figure 2).

**Figure 3:**
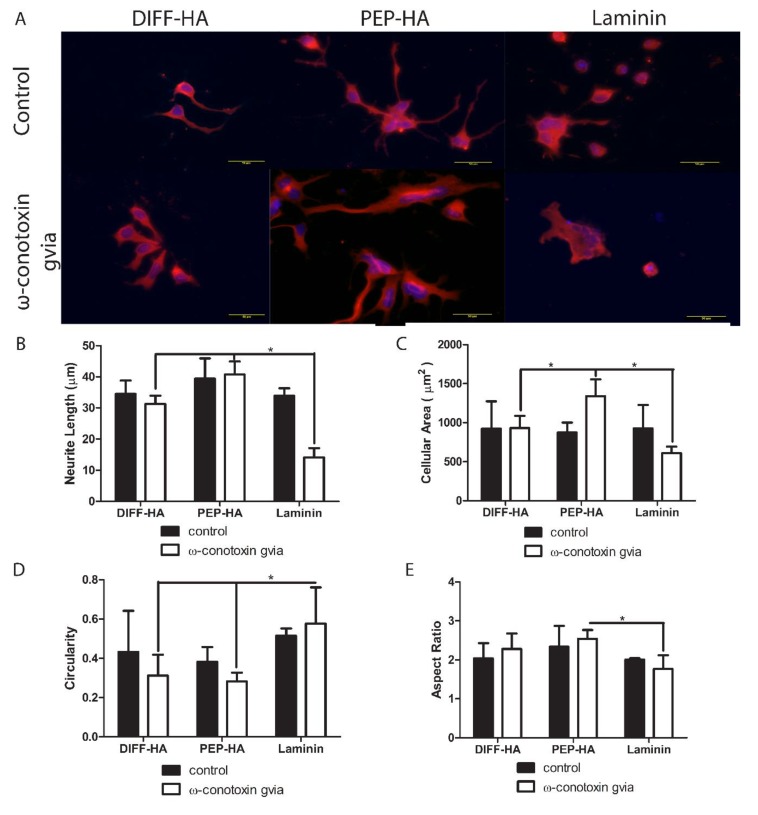
Neuron-specific class III β-tubulin staining (red) with nuclear staining (blue) in hNSC after 1 week of culture on hyaluronic acid-coated tissue culture plastic with (PEP-HA) and without (DIFF-HA) IKVAV and LRE peptide signaling or a laminin-coated control surface (**A**). Small molecule ω-conotoxin GVIA was used to block Ca^2+^ signaling through the CaV2.2 voltage-gated Ca^+^ channels, a known binding target of LRE. Scale bar = 50 µm. neurite length (**B**), cellular area (**C**), cellular circularity (**D**), and cellular aspect ratio (**E**) were measured in the immunoflourescent images. * indicates *p*-value < 0.05 between indicated groups.

**Figure 4:**
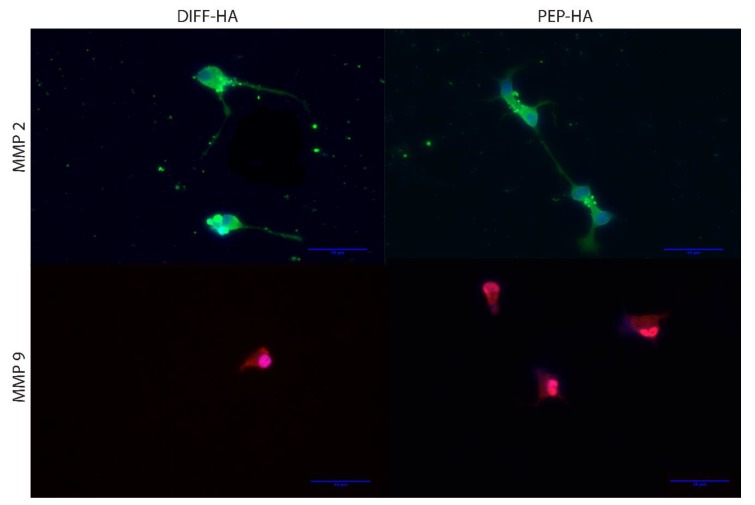
MMP 2 (green) and MMP 9 (red) staining with nuclear counter-stain (blue) after 1 week of neural differentiation culture on hyaluronic-acid-coated tissue culture plastic with (PEP-HA) and without (DIFF-HA) IKVAV and LRE peptide signaling. Scale bar = 50 µm.

**Figure 5:**
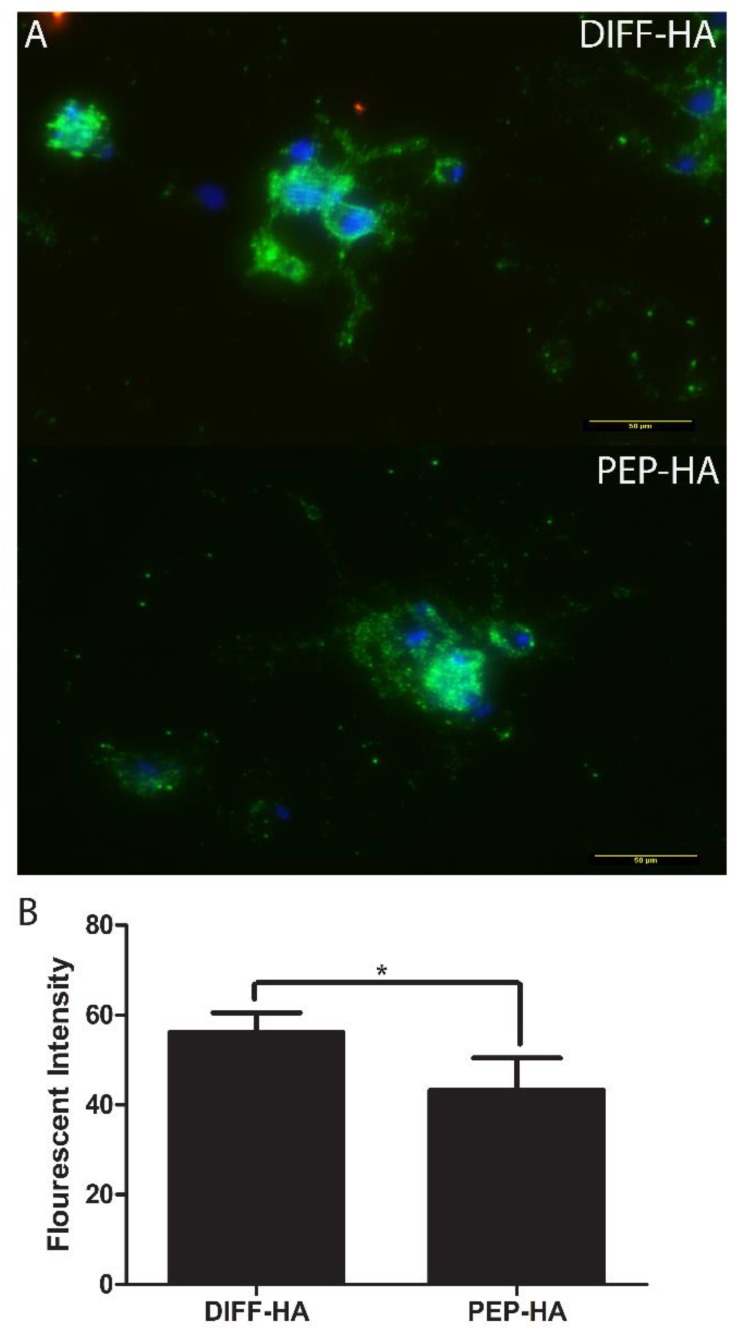
Fibronectin staining (green) staining with nuclear counter-stain (blue) after 1 week of neural differentiation culture on hyaluronic-acid-coated tissue culture plastic with (PEP-HA) and without (DIFF-HA) IKVAV and LRE peptide signaling (**A**). Scale bar = 50 µm. Quantification of fibronectin staining intensity (**B**). * indicates *p* < 0.05 between indicated groups.

After 1 week of neural differentiation culture, the percentage of TUJ1+ cells (DIFF-HA = 99.2 ± 2.6, PEP-HA = 98.8 ± 3.1 and laminin = 100 ± 0 N ≥ 400 cells from three independent samples), average neurite length and standard measures of cellular morphology were found to be similar between test surfaces, but blocking Ca^2+^ flow through Cav2.2 voltage-gated Ca^2+^ channels led to changes in morphology from control conditions on each surface (Figure 3). Staining for MMP 2 and 9 was conducted on DIFF-HA- and PEP-HA-coated surfaces in order to understand how IKVAV and LRE could impact ECM remodeling and axon extension. MMP 2 was expressed down the length of cellular projections of cells cultured on both the DIFF-HA and PEP-HA surfaces (Figure 4). MMP 9 expression was localized on the cell body on both surfaces (Figure 4). Neurons express fibronectin and collagen IV during differentiation [46,47]. Staining for fibronectin was found to be less intense on PEP-HA compared to DIFF-HA surfaces (Figure 5), while collagen IV was not detected on either surface.

## 4. Discussion

Although the effect of ECM properties on cellular behavior is widely studied in other tissues [48,49], their effects on central nervous system behavior have been largely understudied due to the low ECM content in mature CNS tissue [1]. As a result, information regarding the effects of the ECM change on neural cell response is incomplete in the literature. The current study examines the effects of signaling from HA and laminin on hNSC attachment, neurite extension and ECM remodeling. Comparisons of hNSC cultured HA with and without laminin-derived peptide signaling did not promote significant differences in cytoskeletal morphology compared to hNSC cultured on whole laminin (Figure 1 and Figure 3) under standard culture conditions. However, the inclusion of HA was found to increase cellular attachment at 48 h compared to laminin (Figure 2). Previous comparisons of cellular attachment to HA and laminin could not be found in the literature, but comparisons of HA and laminin attachment to other molecules have produced mixed results [15,16,17,18]. Both HA and laminin have been found to increase axon extension separately [3] and in combination [14], but increased neurite extension was not observed in the present study (Figure 3). These inconsistencies in and with the literature could be due to changes in a number of physical and chemical factors between studies that play a role in neural cell attachment and differentiation [50]. In addition, the bioactivity of HA is affected by the amount and type of modification it has undergone [51,52,53,54]. Laminin peptide selection, concentration and confirmation also play a role in their ability to stimulate adhesion and axon extension [28,39,55,56], both of which further complicate comparisons between studies.

Ca^2+^ availability affects attachment and axon extension in a substrate-dependent manner [57,58]. Changes in Ca^2+^ concentration due to altered Cav2.2 transport could activate MMP and lead to the cleavage of cellular receptors for HA [59]. Fewer receptors for HA on the cells would lead to the observed decrease in attachment on HA (Figure 2). Extracellular Ca^2+^ inhibits cell attachment to LRE [34], but does not affect cellular attachment to whole laminin [34,60] or some other laminin-derived peptides [61]. The effects of Ca^2+^ availability on cellular attachment to IKVAV could not be located in the literature. Therefore, IKVAV likely contributed to the increased attachment observed on PEP-HA compared to DIFF-HA (Figure 2). The combination of Cav2.2 voltage-gated Ca^2+^ channel activity and Ca^2+^ concentration is associated with cytoskeletal organization and increased axon extension [62,63,64,65], so it is not unexpected that blocking Cav2.2 Ca^2+^ channel activity changed cytoskeletal organization (Figure 3). Cav2.2 voltage-gated Ca^2+^ channel activity increases the maturation of neuronal morphology [66], while changes in neuronal morphology have been shown to alter Cav2.2 voltage gated Ca^2+^ channel expression and function [67]. Cell size, aspect ratio, and circularity affect neural cell survival [68], lineage choice [69], maturation [70,71,72,73] and axon commitment [74]. Although not often investigated in neural cultures, modulating each of these parameters could substantially impact the efficacy of cell therapy in the CNS. The observed hNSC cytoskeletal organization on the PEP-HA surface is similar to that previously reported in neuronal cell lines overexpressing acetylcholinesterase on laminin surfaces [75]. Acetylcholinesterase contains LRE [32]. The similarities in cytoskeletal changes indicate a potential interaction between IKVAV and LRE signaling. Independent of Cav2.2 Ca^2+^ channel blocking, IKVAV and LRE stimulated integrin signaling interactions with HA signaling pathways that alter cytoskeletal organization [76] and likely contribute to the observed differences in Figure 3.

A previous study found IKVAV and LRE signaling stimulated MMP 2 and 9 expression in a HA matrix [36]. Immunofluorescence staining (Figure 4) indicates MMP 2 likely plays a more direct role in ECM remodeling than MMP 9 in the present system due to its presentation on the cell body and projections. However, MMP 9 regulates Ca^2+^ flow through Cav2.2 voltage-gated Ca^2+^ channels [77] and Ca^2+^ flow through Cav2.2 voltage-gated Ca^2+^ channels stimulates MMP 2 expression [33]. This implies that MMP 9 participates in regulating MMP 2 expression. Future studies will address if this difference in localization contributes to changes in activity or protein level expression.

Differences in MMP activity can lead to changes in ECM content [20]. Laminin, fibronectin and collagen IV expression are associated with neural differentiation [46,47]. However, fibronectin is typically expressed at a higher concentration than the other two proteins [47]. Similar to a previous study [78], the availability of laminin signaling in the matrix reduced the intensity of fibronectin staining (Figure 5). This is important because neurons differentiate between laminin and fibronectin signaling [79]. Laminin signaling better promotes axon extension [79]. Fibronectin slightly inhibits axon extension [80,81]. Reduced fibronectin expression is likely beneficial due to its association with a number of neuro-inflammatory conditions [82] and fibrotic scarring [83,84].

Overall, the data indicate that HA and laminin-derived signaling play complex and commentary roles involving Cav2.2 voltage-gated Ca^2+^ channels to manipulate hNSC behavior and ECM content. Further study is needed to assess the importance of these effects in three dimensional culture and their ability to be developed into useful diagnostic and clinical platforms to aid in the treatment of CNS diseases and injuries.

## Supplementary Materials

The following are available online at https://www.mdpi.com/2079-4983/11/1/15/s1, Figure S1: Phalloidin cytoskeletal staining (red) and vinculin staining (green) with nuclear staining (blue) of hNSC after 48 h of culture in N2B27 maintenance media on laminin-coated tissue culture plastic. Scale bar = 10 µm.

## References

[1] Nicholson C., Sykova E. (1998). Extracellular space structure revealed by diffusion analysis. Trends Neurosci..

[2] Kratochvil M.J., Seymour A.J., Li T.L., Paşca S.P., Kuo C.J., Heilshorn S.C. (2019). Engineered materials for organoid systems. Nat. Rev. Mater..

[3] Long K.R., Huttner W.B. (2019). How the extracellular matrix shapes neural development. Open Biol..

[4] Madl C.M., LeSavage B.L., Dewi R.E., Lampe K.J., Heilshorn S.C. (2019). Matrix Remodeling Enhances the Differentiation Capacity of Neural Progenitor Cells in 3D Hydrogels. Adv. Sci..

[5] Dityatev A., Seidenbecher C.I., Schachner M. (2010). Compartmentalization from the outside: The extracellular matrix and functional microdomains in the brain. Trends Neurosci..

[6] Pan L., Ren Y., Cui F., Xu Q. (2009). Viability and differentiation of neural precursors on hyaluronic acid hydrogel scaffold. J. Neurosci. Res..

[7] Lim H.J., Perera T.H., Wilems T.S., Ghosh S., Zheng Y.Y., Azhdarinia A., Cao Q., Smith Callahan L.A. (2016). Response to di-functionalized hyaluronic acid with orthogonal chemistry grafting at independent modification sites in rodent models of neural differentiation and spinal cord injury. J. Mater. Chem. B.

[8] Zhang Y., Thant A.A., Hiraiwa Y., Naito Y., Sein T.T., Sohara Y., Matsuda S., Hamaguchi M. (2002). A Role for Focal Adhesion Kinase in Hyluronan-Dependent MMP-2 Secretion in a Human Small-Cell Lung Carcinoma Cell Line, QG90. Biochem. Biophys. Res. Commun..

[9] Pedron S., Hanselman J.S., Schroeder M.A., Sarkaria J.N., Harley B.A.C. (2017). Extracellular Hyaluronic Acid Influences the Efficacy of EGFR Tyrosine Kinase Inhibitors in a Biomaterial Model of Glioblastoma. Adv. Healthc. Mater..

[10] Hsu J.-Y.C., McKeon R., Goussev S., Werb Z., Lee J.-U., Trivedi A., Noble-Haeusslein L.J. (2006). Matrix Metalloproteinase-2 Facilitates Wound Healing Events That Promote Functional Recovery after Spinal Cord Injury. J. Neurosci..

[11] Pastrana E., Moreno-Flores M.T., Gurzov E.N., Avila J., Wandosell F., Diaz-Nido J. (2006). Genes Associated with Adult Axon Regeneration Promoted by Olfactory Ensheathing Cells: A New Role for Matrix Metalloproteinase 2. J. Neurosci..

[12] Duchossoy Y., Horvat J.-C., Stettler O. (2001). MMP-Related Gelatinase Activity Is Strongly Induced in Scar Tissue of Injured Adult Spinal Cord and Forms Pathways for Ingrowing Neurites. Mol. Cell. Neurosci..

[13] Bonnans C., Chou J., Werb Z. (2014). Remodelling the extracellular matrix in development and disease. Nat. Rev. Mol. Cell Biol..

[14] Hou S., Xu Q., Tian W., Cui F., Cai Q., Ma J., Lee I.-S. (2005). The repair of brain lesion by implantation of hyaluronic acid hydrogels modified with laminin. J. Neurosci. Methods.

[15] Oohira A., Kushima Y., Tokita Y., Sugiura N., Sakurai K., Suzuki S., Kimata K. (2000). Effects of Lipid-Derivatized Glycosaminoglycans (GAGs), a Novel Probe for Functional Analyses of GAGs, on Cell-to-Substratum Adhesion and Neurite Elongation in Primary Cultures of Fetal Rat Hippocampal Neurons. Arch. Biochem. Biophys..

[16] Zhang Y., Wang L., Zhu J., Hu Y., Xing W., Cheng J. (2012). Real-time monitoring of extracellular matrix-mediated PC12 cell attachment and proliferation using an electronic biosensing device. Biotechnol. Lett..

[17] Ricoult S.G., Goldman J.S., Stellwagen D., Juncker D., Kennedy T.E. (2012). Generation of microisland cultures using microcontact printing to pattern protein substrates. J. Neurosci. Methods.

[18] Calof A.L., Lander A.D. (1991). Relationship between neuronal migration and cell-substratum adhesion: Laminin and merosin promote olfactory neuronal migration but are anti-adhesive. J. Cell Biol..

[19] Lee H., Park J.-W., Kim S.-P., Lo E.H., Lee S.-R. (2009). Doxycycline inhibits matrix metalloproteinase-9 and laminin degradation after transient global cerebral ischemia. Neurobiol. Dis..

[20] Gu Z., Cui J., Brown S., Fridman R., Mobashery S., Strongin A.Y., Lipton S.A. (2005). A Highly Specific Inhibitor of Matrix Metalloproteinase-9 Rescues Laminin from Proteolysis and Neurons from Apoptosis in Transient Focal Cerebral Ischemia. J. Neurosci..

[21] Costa S., Planchenault T., Charriere-Bertrand C., Mouchel Y., Fages C., Juliano S., Lefrançois T., Barlovatz-Meimon G., Tardy M. (2002). Astroglial permissivity for neuritic outgrowth in neuron–astrocyte cocultures depends on regulation of laminin bioavailability. Glia.

[22] Khan K.M.F., Falcone D.J. (2000). Selective Activation of MAPKerk1/2 by Laminin-1 Peptide α1:Ser2091–Arg2108 Regulates Macrophage Degradative Phenotype. J. Biol. Chem..

[23] Yamada Y., Hozumi K., Nomizu M. (2011). Construction and activity of a synthetic basement membrane with active laminin peptides and polysaccharides. Chemistry.

[24] Patel R., Santhosh M., Dash J.K., Karpoormath R., Jha A., Kwak J., Patel M., Kim J.H. (2019). Ile-Lys-Val-ala-Val (IKVAV) peptide for neuronal tissue engineering. Polym. Adv. Technol..

[25] Tashiro K., Sephel G.C., Weeks B., Sasaki M., Martin G.R., Kleinman H.K., Yamada Y. (1989). A synthetic peptide containing the IKVAV sequence from the A chain of laminin mediates cell attachment, migration, and neurite outgrowth. J. Biol. Chem..

[26] Freitas V.M., Vilas-Boas V.F., Pimenta D.C., Loureiro V., Juliano M.A., Carvalho M.R., Pinheiro J.J.V., Camargo A.C.M., Moriscot A.S., Hoffman M.P. (2007). SIKVAV, a Laminin α1-Derived Peptide, Interacts with Integrins and Increases Protease Activity of a Human Salivary Gland Adenoid Cystic Carcinoma Cell Line through the ERK 1/2 Signaling Pathway. Am. J. Pathol..

[27] Que R.A., Arulmoli J., Da Silva N.A., Flanagan L.A., Wang S.W. (2018). Recombinant collagen scaffolds as substrates for human neural stem/progenitor cells. J. Biomed. Mater. Res. Part A.

[28] Li X., Liu X., Josey B., Chou C.J., Tan Y., Zhang N., Wen X. (2014). Short Laminin Peptide for Improved Neural Stem Cell Growth. Stem Cells Transl. Med..

[29] Pan L., North H.A., Sahni V., Jeong S.J., McGuire T.L., Berns E.J., Stupp S.I., Kessler J.A. (2014). beta1-Integrin and integrin linked kinase regulate astrocytic differentiation of neural stem cells. PLoS ONE.

[30] Lam J., Carmichael S.T., Lowry W.E., Segura T. (2015). Design of experiments methodology to optimize hydrogel for iPSC-NPC culture. Adv. Healthc. Mater..

[31] Farrukh A., Ortega F., Fan W., Marichal N., Paez J.I., Berninger B., Campo A.d., Salierno M.J. (2017). Bifunctional Hydrogels Containing the Laminin Motif IKVAV Promote Neurogenesis. Stem Cell Rep..

[32] Johnson G., Moore S.W. (2013). The Leu-Arg-Glu (LRE) adhesion motif in proteins of the neuromuscular junction with special reference to proteins of the carboxylesterase/cholinesterase family. Comp. Biochem. Physiol. Part D Genom. Proteom..

[33] Stettner M., Dehmel T., Mausberg A.K., Kohne A., Rose C.R., Kieseier B.C. (2011). Levetiracetam exhibits protective properties on rat Schwann cells in vitro. J. Peripher. Nerv. Syst. JPNS.

[34] Hunter D.D., Cashman N., Morris-Valero R., Bulock J.W., Adams S.P., Sanes J.R. (1991). An LRE (leucine-arginine-glutamate)-dependent mechanism for adhesion of neurons to S-laminin. J. Neurosci. Off. J. Soc. Neurosci..

[35] Nishimune H., Sanes J.R., Carlson S.S. (2004). A synaptic laminin-calcium channel interaction organizes active zones in motor nerve terminals. Nature.

[36] Perera T.H., Howell S.M., Smith Callahan L.A. (2019). Manipulation of Extracellular Matrix Remodeling and Neurite Extension by Mouse Embryonic Stem Cells using IKVAV and LRE Peptide Tethering in Hyaluronic Acid Matrices. Biomacromolecules.

[37] Lim H.J., Khan Z., Wilems T.S., Lu X., Perera T.H., Kurosu Y.E., Ravivarapu K.T., Mosley M.C., Smith Callahan L.A. (2017). Human Induced Pluripotent Stem Cell Derived Neural Stem Cell Survival and Neural Differentiation on Polyethylene Glycol Dimethacrylate Hydrogels Containing a Continuous Concentration Gradient of N-Cadherin Derived Peptide His-Ala-Val-Asp-Ile. ACS Biomater. Sci. Eng..

[38] Lim H.J., Mosley M.C., Kurosu Y., Smith Callahan L.A. (2017). Concentration dependent survival and neural differentiation of murine embryonic stem cells cultured on polyethylene glycol dimethacrylate hydrogels possessing a continuous concentration gradient of n-cadherin derived peptide His-Ala-Val-Asp-Lle. Acta Biomater..

[39] Lim H.J., Khan Z., Lu X., Perera T.H., Wilems T.S., Ravivarapu K.T., Smith Callahan L.A. (2018). Mechanical stabilization of proteolytically degradable polyethylene glycol dimethacrylate hydrogels through peptide interaction. Acta Biomater..

[40] Smith Callahan L.A., Ganios A.M., Childers E.P., Weiner S.D., Becker M.L. (2013). Primary human chondrocyte extracellular matrix formation and phenotype maintenance using RGD-derivatized PEGDM hydrogels possessing a continuous Young’s modulus gradient. Acta Biomater..

[41] Karlsson M., Björnsson S., Iozzo R.V. (2001). Quantitation of Proteoglycans in Biological Fluids Using Alcian Blue. Proteoglycan Protocols.

[42] Li S., Xue H., Wu J., Rao M.S., Kim D.H., Deng W., Liu Y. (2015). Human Induced Pluripotent Stem Cell NEUROG2 Dual Knockin Reporter Lines Generated by the CRISPR/Cas9 System. Stem Cells Dev..

[43] Long B., Li S., Xue H., Sun L., Kim D.H., Liu Y. (2017). Effects of Propofol Treatment in Neural Progenitors Derived from Human-Induced Pluripotent Stem Cells. Neural Plast..

[44] Mosley M.C., Lim H.J., Chen J., Yang Y.-H., Li S., Liu Y., Smith Callahan L.A. (2017). Neurite extension and neuronal differentiation of human induced pluripotent stem cell derived neural stem cells on polyethylene glycol hydrogels containing a continuous Young’s Modulus gradient. J. Biomed. Mater. Res. Part A.

[45] Wilems T.S., Lu X., Kurosu Y.E., Khan Z., Lim H.J., Smith Callahan L.A. (2017). Effects of free radical initiators on polyethylene glycol dimethacrylate hydrogel properties and biocompatibility. J. Biomed. Mater. Res. Part A.

[46] Sart S., Ma T., Li Y. (2014). Extracellular matrices decellularized from embryonic stem cells maintained their structure and signaling specificity. Tissue Eng. Part A.

[47] Bento A.R., Quelhas P., Oliveira M.J., Pego A.P., Amaral I.F. (2017). Three-dimensional culture of single embryonic stem-derived neural/stem progenitor cells in fibrin hydrogels: Neuronal network formation and matrix remodelling. J. Tissue Eng. Regen. Med..

[48] Smith L.A., Liu X., Ma P.X. (2008). Tissue engineering with nano-fibrous scaffolds. Soft Matter.

[49] Smith Callahan L.A. (2018). Gradient Material Strategies for Hydrogel Optimization in Tissue Engineering Applications. High Throughput.

[50] Farrukh A., Zhao S., del Campo A. (2018). Microenvironments Designed to Support Growth and Function of Neuronal Cells. Front. Mater..

[51] Eng D., Caplan M., Preul M., Panitch A. (2010). Hyaluronan scaffolds: A balance between backbone functionalization and bioactivity. Acta Biomater..

[52] Lord M.S., Pasqui D., Barbucci R., Milthorpe B.K. (2009). Protein adsorption on derivatives of hyaluronic acid and subsequent cellular response. J. Biomed. Mater. Res. Part A.

[53] Bencherif S.A., Srinivasan A., Horkay F., Hollinger J.O., Matyjaszewski K., Washburn N.R. (2008). Influence of the degree of methacrylation on hyaluronic acid hydrogels properties. Biomaterials.

[54] Köwitsch A., Yang Y., Ma N., Kuntsche J., Mäder K., Groth T. (2011). Bioactivity of immobilized hyaluronic acid derivatives regarding protein adsorption and cell adhesion. Biotechnol. Appl. Biochem..

[55] Yang Y.H., Khan Z., Ma C., Lim H.J., Smith Callahan L.A. (2015). Optimization of adhesive conditions for neural differentiation of murine embryonic stem cells using hydrogels functionalized with continuous Ile-Lys-Val-Ala-Val concentration gradients. Acta Biomater..

[56] Tong Y.W., Shoichet M.S. (2001). Enhancing the neuronal interaction on fluoropolymer surfaces with mixed peptides or spacer group linkers. Biomaterials.

[57] Moran D. (1984). Fluorescent localization of calcium at sites of cell attachment and spreading. J. Exp. Zool..

[58] Henley J., Poo M.-m. (2004). Guiding neuronal growth cones using Ca^2+^ signals. Trends Cell Biol..

[59] Nagano O., Murakami D., Hartmann D., de Strooper B., Saftig P., Iwatsubo T., Nakajima M., Shinohara M., Saya H. (2004). Cell–matrix interaction via CD44 is independently regulated by different metalloproteinases activated in response to extracellular Ca^2+^ influx and PKC activation. J. Cell Biol..

[60] Lallier T., Bronner-Fraser M. (1992). Alpha 1 beta 1 integrin on neural crest cells recognizes some laminin substrata in a Ca(2+)-independent manner. J. Cell Biol..

[61] Yamada Y., Hozumi K., Katagiri F., Kikkawa Y., Nomizu M. (2013). Laminin-111-derived peptide-hyaluronate hydrogels as a synthetic basement membrane. Biomaterials.

[62] Dombert B., Balk S., Lüningschrör P., Moradi M., Sivadasan R., Saal-Bauernschubert L., Jablonka S. (2017). BDNF/trkB Induction of Calcium Transients through Ca(v)2.2 Calcium Channels in Motoneurons Corresponds to F-actin Assembly and Growth Cone Formation on β2-Chain Laminin (221). Front. Mol. Neurosci..

[63] Kerstein P.C., Patel K.M., Gomez T.M. (2017). Calpain-Mediated Proteolysis of Talin and FAK Regulates Adhesion Dynamics Necessary for Axon Guidance. J. Neurosci..

[64] Ould-yahoui A., Tremblay E., Sbai O., Ferhat L., Bernard A., Charrat E., Gueye Y., Lim N.H., Brew K., Risso J.J. (2009). A new role for TIMP-1 in modulating neurite outgrowth and morphology of cortical neurons. PLoS ONE.

[65] Pravettoni E., Bacci A., Coco S., Forbicini P., Matteoli M., Verderio C. (2000). Different localizations and functions of L-type and N-type calcium channels during development of hippocampal neurons. Dev. Biol..

[66] Marques J.M., Rodrigues R.J., Valbuena S., Rozas J.L., Selak S., Marin P., Aller M.I., Lerma J. (2013). CRMP2 Tethers Kainate Receptor Activity to Cytoskeleton Dynamics during Neuronal Maturation. J. Neurosci..

[67] Wang W., Zhong D., Lin Y., Fan R., Hou Z., Cao X., Ren Y. (2018). Responsiveness of voltage-gated calcium channels in SH-SY5Y human neuroblastoma cells on micropillar substrates. J. Biomater. Sci. Polym. Ed..

[68] Shin H.Y., Pfaff K.L., Davidow L.S., Sun C., Uozumi T., Yanagawa F., Yamazaki Y., Kiyota Y., Rubin L.L. (2018). Using Automated Live Cell Imaging to Reveal Early Changes during Human Motor Neuron Degeneration. Eneuro.

[69] Qi L., Li N., Huang R., Song Q., Wang L., Zhang Q., Su R., Kong T., Tang M., Cheng G. (2013). The Effects of Topographical Patterns and Sizes on Neural Stem Cell Behavior. PLoS ONE.

[70] Kang S., Chen X., Gong S., Yu P., Yau S., Su Z., Zhou L., Yu J., Pan G., Shi L. (2017). Characteristic analyses of a neural differentiation model from iPSC-derived neuron according to morphology, physiology, and global gene expression pattern. Sci. Rep..

[71] Tan K.K.B., Tann J.Y., Sathe S.R., Goh S.H., Ma D., Goh E.L.K., Yim E.K.F. (2015). Enhanced differentiation of neural progenitor cells into neurons of the mesencephalic dopaminergic subtype on topographical patterns. Biomaterials.

[72] Ankam S., Lim C.K., Yim E.K.F. (2015). Actomyosin contractility plays a role in MAP2 expression during nanotopography-directed neuronal differentiation of human embryonic stem cells. Biomaterials.

[73] Kono S., Yamamoto H., Kushida T., Hirano-Iwata A., Niwano M., Tanii T. (2016). Live-Cell, Label-Free Identification of GABAergic and Non-GABAergic Neurons in Primary Cortical Cultures Using Micropatterned Surface. PLoS ONE.

[74] Yamamoto H., Demura T., Morita M., Banker G.A., Tanii T., Nakamura S. (2012). Differential neurite outgrowth is required for axon specification by cultured hippocampal neurons. J. Neurochem..

[75] Sperling L.E., Klaczinski J., Schütz C., Rudolph L., Layer P.G. (2012). Mouse Acetylcholinesterase Enhances Neurite Outgrowth of Rat R28 Cells through Interaction with Laminin-1. PLoS ONE.

[76] Chopra A., Murray M.E., Byfield F.J., Mendez M.G., Halleluyan R., Restle D.J., Raz-Ben Aroush D., Galie P.A., Pogoda K., Bucki R. (2014). Augmentation of integrin-mediated mechanotransduction by hyaluronic acid. Biomaterials.

[77] Brittain J.M., Piekarz A.D., Wang Y., Kondo T., Cummins T.R., Khanna R. (2009). An Atypical Role for Collapsin Response Mediator Protein 2 (CRMP-2) in Neurotransmitter Release via Interaction with Presynaptic Voltage-gated Calcium Channels. J. Biol. Chem..

[78] Stabenfeldt S.E., Munglani G., García A.J., LaPlaca M.C. (2010). Biomimetic microenvironment modulates neural stem cell survival, migration, and differentiation. Tissue Eng. Part A.

[79] Myers J.P., Santiago-Medina M., Gomez T.M. (2011). Regulation of axonal outgrowth and pathfinding by integrin-ECM interactions. Dev. Neurobiol..

[80] Deister C., Aljabari S., Schmidt C.E. (2007). Effects of collagen 1, fibronectin, laminin and hyaluronic acid concentration in multi-component gels on neurite extension. J. Biomater. Sci. Polym. Ed..

[81] Giordano C., Poiana G., Augusti-Tocco G., Biagioni S. (2007). Acetylcholinesterase modulates neurite outgrowth on fibronectin. Biochem. Biophys. Res. Commun..

[82] Willis C.M., Crocker S.J., Travascio F. (2016). The Mosaic of Extracellular Matrix in the Central Nervous System as a Determinant of Glial Heterogeneity. Composition and Function of the Extracellular Matrix in the Human Body.

[83] Cooper J.G., Jeong S.J., McGuire T.L., Sharma S., Wang W., Bhattacharyya S., Varga J., Kessler J.A. (2018). Fibronectin EDA forms the chronic fibrotic scar after contusive spinal cord injury. Neurobiol. Dis..

[84] Zhu Y., Soderblom C., Trojanowsky M., Lee D.-H., Lee J.K. (2015). Fibronectin Matrix Assembly after Spinal Cord Injury. J. Neurotrauma.

