# Supplementary material for: Effect of Laminin Derived Peptides IKVAV and LRE Tethered to Hyaluronic Acid on hiPSC Derived Neural Stem Cell Morphology, Attachment and Neurite Extension
Source: J Funct Biomater. 2020 Mar 6;11(1):15. doi: 10.3390/jfb11010015 (PMC7151619; doi:10.3390/jfb11010015)
Supplement: Supplementary file 1 [file jfb-11-00015-s001.pdf]

Supplementary Materials

Effect of Laminin Derived Peptides IKVAV and LRE Tethered to Hyaluronic Acid on hiPSC Derived Neural Stem Cell Morphology, Attachment and Neurite Extension

T. Hiran Perera [1,2], Xi Lu [1,2] and Laura A Smith Callahan [1,2,3,*]

1 Vivian L. Smith Department of Neurosurgery, McGovern Medical School at the University of Texas Health Science Center at Houston McGovern Medical School, Houston, TX 77030, USA; Thuduwage.H.Perera@uth.tmc.edu (T.H.P.); xi.lu@uth.tmc.edu (X.L.)

2 Center for Stem Cell and Regenerative Medicine, Brown Foundation Institute of Molecular Medicine, McGovern Medical School at the University of Texas Health Science Center at Houston, Houston, TX 77030, USA

3 Graduate School of Biomedical Sciences, MD Anderson Cancer Center UTHealth, Houston, TX 77030, USA

* Correspondence: laura.a.smithcallahan@uth.tmc.edu; Tel.: 1-713-500-3431.

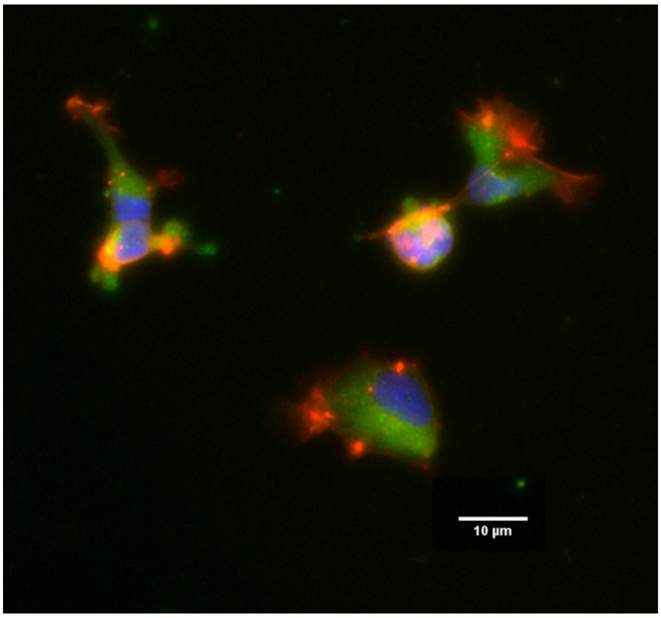

Figure S1: Phalloidin cytoskeletal staining (red) and vinculin staining (green) with nuclear staining (blue) of hNSC after 48 h of culture in N2B27 maintenance media on laminin coated tissue culture plastic. Scale bar = 10 µm.
